# The Glypican proteoglycans show intrinsic interactions with Wnt-3a in human prostate cancer cells that are not always associated with cascade activation

**DOI:** 10.1186/s12860-021-00361-x

**Published:** 2021-05-04

**Authors:** Gabrielle Ferrante Alves de Moraes, Eduardo Listik, Giselle Zenker Justo, Carolina Meloni Vicente, Leny Toma

**Affiliations:** 1 Departamento de Bioquímica (Campus São Paulo), Universidade Federal de São Paulo, Rua Três de Maio, P.O. Box: 04044-020, São Paulo, SP 100 Brazil; 2 Departamento de Ciências Biológicas (Campus Diadema), Universidade Federal de São Paulo, Rua Três de Maio, P.O. Box: 04044-020, São Paulo, SP 100 Brazil

**Keywords:** Proteoglycans, Glypicans, Wnt signaling, Prostate cancer, PC-3 cells

## Abstract

**Background:**

Prostate cancer occurs through multiple steps until advanced metastasis. Signaling pathways studies can result in the identification of targets to interrupt cancer progression. Glypicans are cell surface proteoglycans linked to the membrane through glycosylphosphatidylinositol. Their interaction with specific ligands has been reported to trigger diverse signaling, including Wnt. In this study, prostate cancer cell lines PC-3, DU-145, and LNCaP were compared to normal prostate RWPE-1 cell line to investigate glypican family members and the activation of the Wnt signaling pathway.

**Results:**

Glypican-1 (GPC1) was highly expressed in all the examined cell lines, except for LNCaP, which expressed glypican-5 (GPC5). The subcellular localization of GPC1 was detected on the cell surface of RWPE-1, PC-3, and DU-145 cell lines, while GPC5 suggested cytoplasm localization in LNCaP cells. Besides glypican, flow cytometry analysis in these prostate cell lines confirmed the expression of Wnt-3a and unphosphorylated β-catenin. The co-immunoprecipitation assay revealed increased levels of binding between Wnt-3a and glypicans in cancer cells, suggesting a relationship between these proteoglycans in this pathway. A marked increase in nuclear β-catenin was observed in tumor cells. However, only PC-3 cells demonstrated activation of canonical Wnt signaling, according to the TOPFLASH assay.

**Conclusions:**

GPC1 was the majorly expressed gene in all the studied cell lines, except for LNCaP, which expressed GPC5. We assessed by co-immunoprecipitation that these GPCs could interact with Wnt-3a. However, even though nuclear β-catenin was found increased in the prostate cancer cells (i.e., PC-3, DU-145 and LNCaP), activation of Wnt pathway was only found in PC-3 cells. In these PC-3 cells, GPC1 and Wnt-3a revealed high levels of colocalization, as assessed by confocal microscopy studies. This suggests a localization at the cellular surface, where Frizzled receptor is required for downstream activation. The interaction of Wnt-3a with GPCs in DU-145 and LNCaP cells, which occurs in absence of Wnt signaling activation, requires further studies. Once non-TCF-LEF proteins can also bind β-catenin, another signaling pathway may be involved in these cells with regulatory function.

**Supplementary Information:**

The online version contains supplementary material available at 10.1186/s12860-021-00361-x.

## Background

According to an annual study from the American Cancer Society, prostate cancer is the most common non-skin malignancy in men and is the second leading cause of cancer-specific deaths among men throughout the world. In the year 2017, more than 160,000 new cases were registered in the United States alone [1]. The underlying causes of prostate cancer are not yet fully understood; however, some associated risk factors have been identified, such as age, ethnicity, genetics, and diet. The risks for this type of cancer increase with age, and prostate cancer rarely occurs in men younger than 50 years of age [2]. Recent studies have shown that advanced and metastatic prostate cancer can be stimulated by the deregulation of multiple signaling pathways that act through the androgen receptor [3, 4].

Proteoglycans (PGs) are macromolecules that are formed by a core protein and a different number of covalently linked polysaccharide chains, called glycosaminoglycans (GAGs). GAGs are polymers composed of disaccharides subunits formed by a hexosamine, such as N-acetylglucosamine or N-acetylgalactosamine, and a uronic acid, such as glucuronic acid or its epimer-iduronic acid. PGs are vital components during organism development, cell proliferation, adhesion, migration, and cellular signaling [5]. Not only are PGs important factors during physiological conditions, but they also participate in many pathological aspects of cancer, including prostate cancer.

Glypicans are heparan sulfate (HS) PGs (HSPGs), which are inserted into the cell membrane by a glycosylphosphatidylinositol (GPI) moiety. Six glypican members (GPC1–6) have been described in humans, which differ among their core protein compositions. The presence of HS chains at the carboxyl terminus, close to the cell membrane, suggests that GPCs interact with signaling molecules, such as growth factor receptors and ligands. GPCs may appear in lipid rafts, domains that facilitate communication between proteins for cell signaling mechanisms. Moreover, specific GPCs have previously been shown to be essential for the regulation of signaling molecules, including Hedgehog proteins (Hhs), fibroblast growth factors (FGFs), Wnts, transforming growth factors (TGFs), epidermal growth factors (EGFs) and bone morphogenetic proteins (BMPs) [6–8]. Additionally, the role of GPCs in prostate cancer has been previously established. GPC1 has been proposed to represent a biomarker for prostate cancer, with better efficiency than the prostate-specific antigens (PSA) [9]. In contrast, GPC5 protein expression levels examined by immunohistochemistry, are reduced in prostate cancer tissues compared with normal tissues, indicating that GPC5 could represent an additional prostate cancer biomarker [10].

Various Wnt ligands exist (Wnt1–19), which are secreted into the extracellular medium. The canonical Wnt pathway relies on the translocation of β-catenin to the nucleus, whereas the non-canonical Wnt signaling pathway does not include the β-catenin interaction. β-catenin is constitutively degraded after phosphorylation by a destruction complex, which includes adenomatous polyposis coli protein (APC), casein kinase 1 (CK1), Axin1 and glycogen synthase kinase-3 (GSK3). During the activation of the canonical signaling pathway, specific Wnt ligands (such as Wnt-3a) interact with Frizzled and low-density liporeceptor (LDL)-receptor related protein (LRP)5/6 to inactivate the destruction complex, enabling the translocation of unphosphorylated β-catenin to the nucleus, where it interacts with transcription factors, such as T-cell factor-lymphoid enhancer-binding factor 1 (TCF-LEF) [11, 12]. Wnt signaling is dysregulated in several human cancers, including prostate cancer. Therefore, canonical Wnt signaling appears to be a factor that contributes to prostate cancer progression [13].

The dysregulation and abnormal behavior of the Wnt pathway are prevalent themes in cancer biology and the study of GPCs. A better understanding of the interactions between these molecules could provide relevant information regarding oncologic treatments and prognostics. Thus, the primary objective of this study was to investigate the relationship between GPCs and Wnt signaling in prostate cancer cells. This study can offer a great perspective on how the different GPCs and Wnt signaling components behave during prostate cancer and how they are involved in this disease.

## Results

### The glypican-1 and -5 are predominantly expressed in prostate cancer cells

**Fig. 1:**
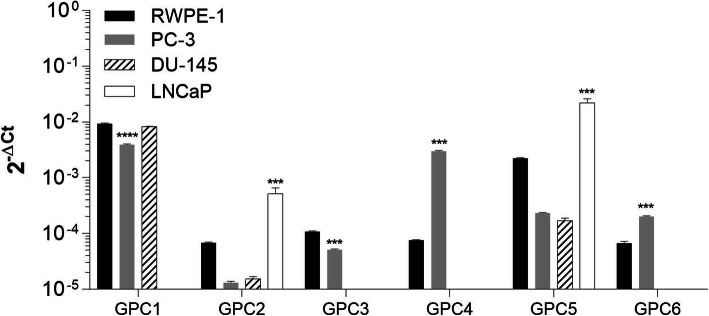
RWPE-1, PC-3, DU-145, and LNCaP mRNA levels for all glypican family members. The expression levels were assessed by RT-qPCR and normalized to the expression levels of GAPDH. Data are plotted as the mean ± s.e.m. of the 2^-ΔCT^ values, and three independent experiments were performed. Expression levels in prostate cancer cells were compared with those in the normal prostate cell line (RWPE-1) using Dunnett’s test. *** *p* < 0.001; **** *p* < 0.0001

The gene expression of the GPC family members was examined in RWPE-1, PC-3, DU-145, and LNCaP cell lines by RT-qPCR (Fig. 1). PC-3 and DU-145 cells are derived from bone and brain metastasis, respectively, they have high metastatic potential and androgen-refractory response. Otherwise, LNCaP cells from lymph node metastasis are androgen-sensitive and present low-metastatic properties [14]. GPC1 and GPC5 were predominantly expressed in the normal prostate cell line RWPE-1, whereas GPC1 was the primary member observed in the prostate cancer cell lines PC-3 and DU-145. However, unlike the other cell lines, the LNCaP cell line did not express GPC1 but did express high levels of GPC5.

From the collected data, a pattern of gene expression cannot be established for GPCs when comparing normal and cancer prostate cells. Although some glypican members, such as GPC1, 2, 3, and 5, appeared to be expressed at lower levels in tumor cells compared to RWPE-1 cells, others seemed to be expressed at higher levels in PC-3 cells, such as GPC4 and 6. In contrast, LNCaP cells demonstrated opposite responses for GPC2 and 5, with higher gene expression levels of these members than RWPE-1. These results refer to mRNA levels, which does not necessarily reflect the levels of protein of each glypican family member.

### Glypican-1 is present in RWPE-1, PC-3, and DU-145 cell lines, while glypican-5 is present in LNCaP

**Fig. 2:**
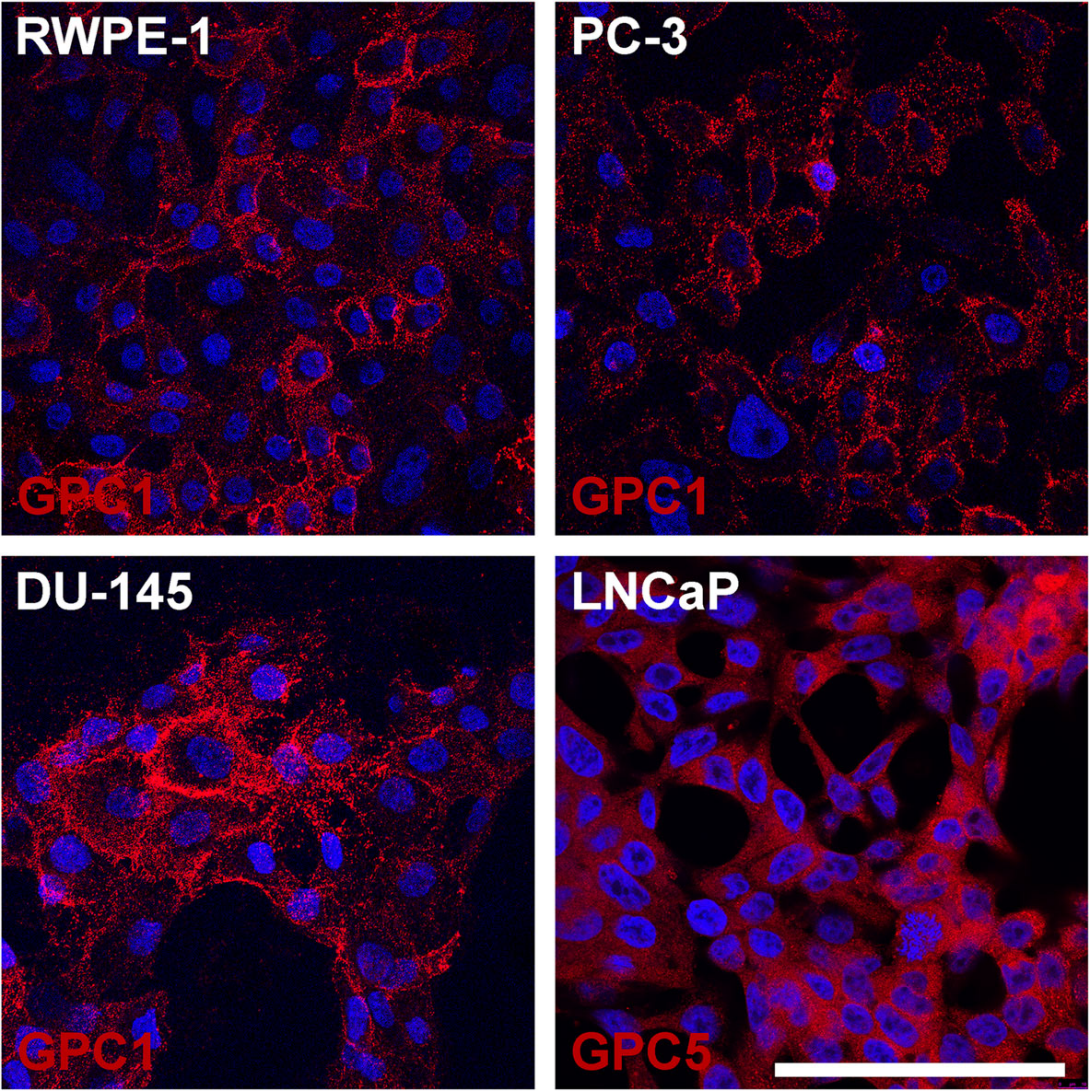
Immunofluorescence assay to investigate selected GPCs’ localization in RWPE-1, PC-3, DU-145, and LNCaP cells. Cell staining was assessed by confocal microscopy. The different glypicans are stained in red, and nuclei, stained with DAPI, are in blue. The scale bar equals 100 μm

After investigating the gene expression levels of the different GPCs using RT-qPCR, we proceeded to perform protein analyses for GPC1 and 5. First, immunofluorescence analyses of GPC1 were performed in RWPE-1, PC-3, and DU-145 cell lines, whereas immunofluorescence analysis of GPC5 was explicitly performed in LNCaP cells (Fig. 2). In RWPE-1, PC-3, and DU-145 cells, GPC1 was found at the outer region of the cell, indicating a possible surface location. DU-145 cells also presented some granulations inward from the membrane. Interestingly, in the LNCaP cell line, the GPC5 presented a diffuse staining, indicating a location predominantly in the cytoplasm.

### Wnt-3a signaling is altered in prostate cancer cells, which express GPCs differently

**Fig. 3:**
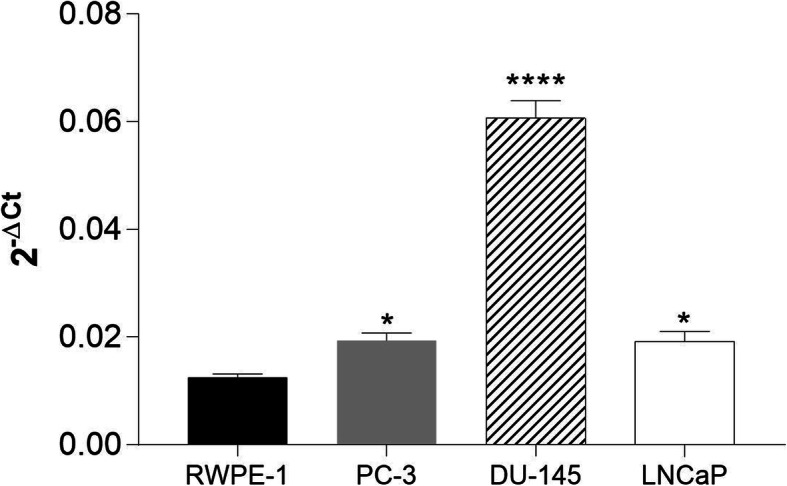
β-catenin mRNA levels in RWPE-1, PC-3, DU-145, and LNCaP cells. Gene expression levels were quantified using RT-qPCR and normalized to the levels of GAPDH expression. Data are plotted as the mean ± s.e.m. of the 2^-ΔCT^ values, and three independent experiments were performed. Expression levels in prostate cancer cells were compared with those in the normal prostate cell line (RWPE-1) using Dunnett’s test. * *p* < 0.05; **** *p* < 0.0001

To study the canonical Wnt signaling in prostate cells, we began by analyzing the expression of the β-catenin gene using RT-qPCR (Fig. 3). Tumor cells expressed higher levels of β-catenin than normal prostate cells (i.e., RWPE-1 cells).

**Fig. 4:**
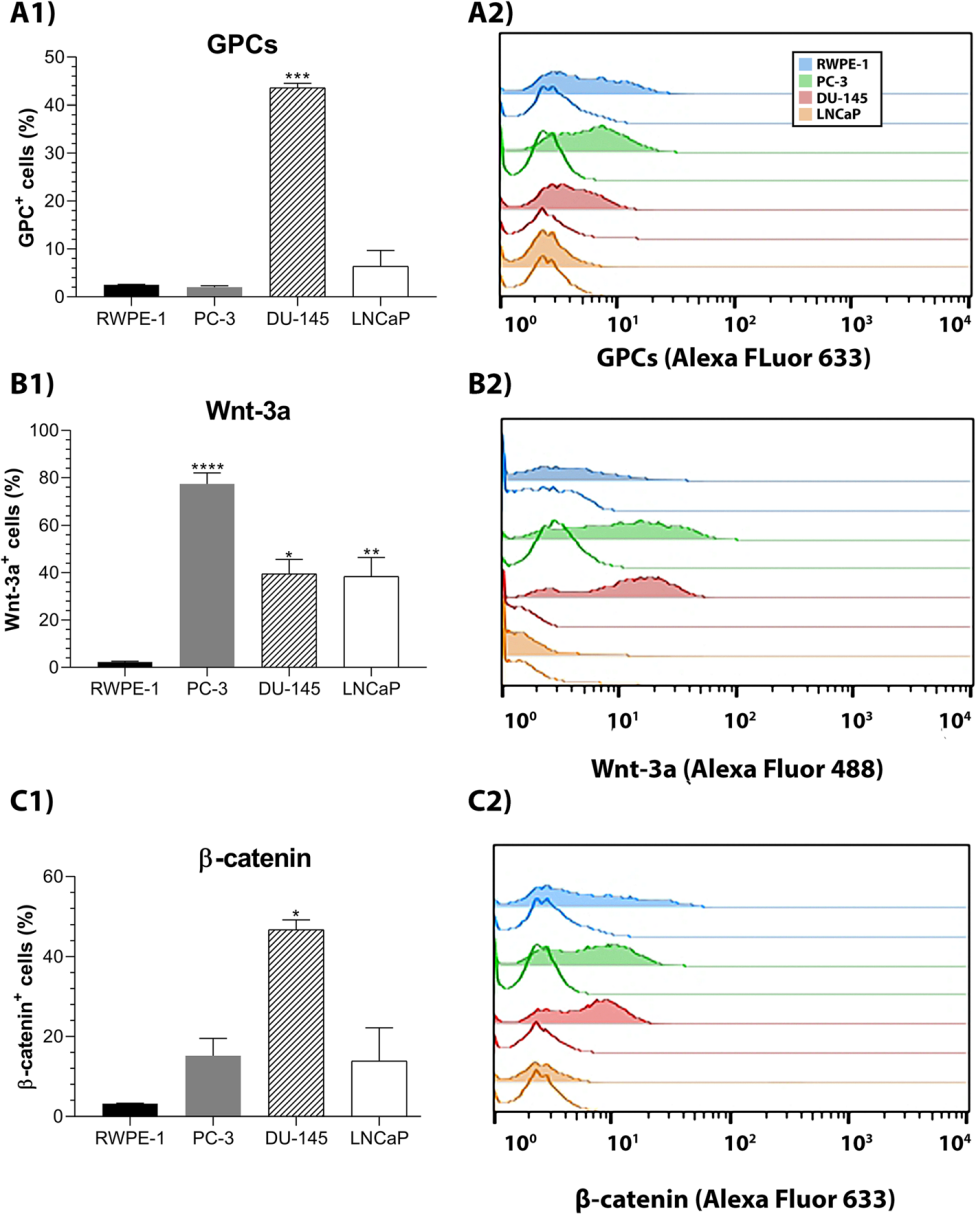
Flow cytometry analysis of GPCs, Wnt-3a and β-catenin in prostate cancer cell lines. **a** GPCs, **b** Wnt-3a, and (**c**) β-catenin were analyzed in RWPE-1, PC-3, DU-145 and LNCaP. GPC1 was assessed in RWPE-1 (blue), PC-3 (green), and DU-145 cells (red), while GPC5 was assessed in LNCaP cells (orange). (1) The positively stained cells for each protein were quantified and plotted. (2) The histograms reveal the distributions of unstained control (unfilled) and stained experimental assessments (filled) of GPCs, Wnt-3a, and β-catenin. Data are plotted as the mean ± s.e.m. Prostate cancer cells were compared to RWPE-1 cells using Dunnett’s test. (*n* = 2–8). * *p* < 0.05; ** *p* < 0.01; *** *p* < 0.001; **** *p* < 0.0001)

Previous data from our group demonstrated the relevance of Wnt-3A for the tumorigenicity of PC-3 and DU-145 cell lines [15]. We then performed flow cytometry analyses for β-catenin and Wnt-3a and correlated these data with GPC1 or − 5 protein contents (Fig. 4). By this assay, DU-145 cells were observed to express higher levels of GPC than any other cell line (Fig. 4A1), which was directly linked to the high levels of β-catenin gene and protein expression in these cells (Fig. 4C1). All three prostate cancer cell lines, PC-3, DU-145, and LNCaP, expressed higher levels of Wnt-3a than the normal cell line, RWPE-1 (Fig. 4B1).

**Fig. 5:**
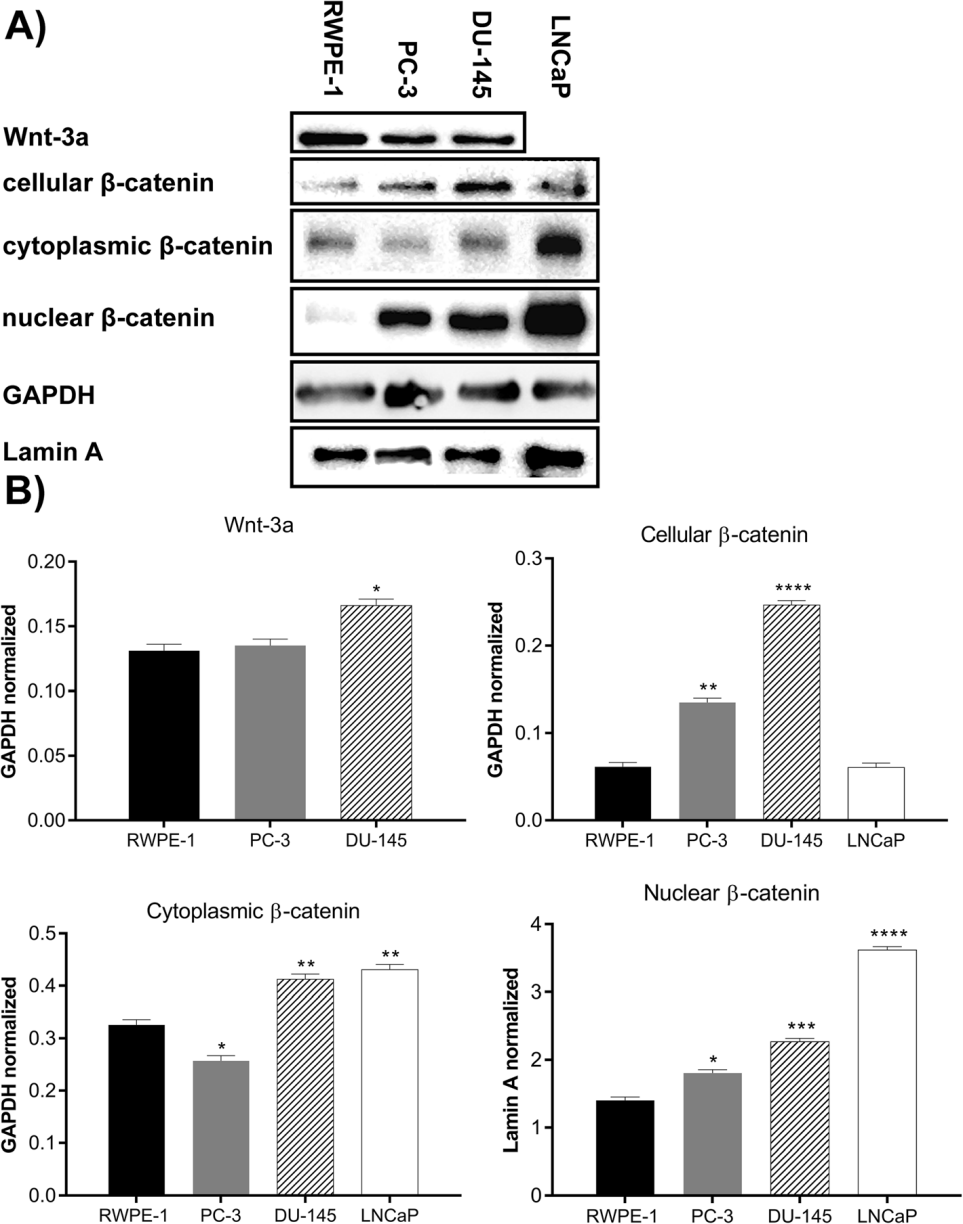
Immunoblot assays of RWPE-1, PC-3, DU-145, and LNCaP cells, evaluating Wnt-3a, and β-catenin. LNCaP cells were not analyzed using the Wnt-3a antibody. **a** Immunoblot results for Wnt-3a, cellular, cytoplasmic and nuclear β-catenin for RWPE-1, PC-3, DU-145, and LNCaP. Housekeeping proteins such as GAPDH and Lamin A were also investigated. **b** Densitometric analyses of the immunoblots, where data are normalized against GAPDH, except for nuclear β-catenin, which was normalized against Lamin A. Data are plotted as the mean ± s.e.m., and two independent experiments were performed. Prostate cancer cells were compared to RWPE-1 cells using Dunnett’s test. * *p* < 0.05; ** *p* < 0.01; *** *p* < 0.001; **** *p* < 0.0001

To further validate our findings, immunoblotting was performed (Fig. 5a) using whole cell extract. After the quantification of protein levels, and normalization against the expression level of GAPDH (Fig. 5b), DU-145 cells demonstrated a higher protein expression level of Wnt-3a than the other cell lines. PC-3 and DU-145 showed elevated levels of cellular β-catenin (Fig. 5b) compared with the other cells. Moreover, we examined whether β-catenin was predominantly localized in the nucleus or the cytoplasm and found that, in prostate cancer cells, β-catenin was significantly concentrated in the cell nucleus compared with a more cytoplasmic proportion in RWPE-1 cells (Fig. 5b).

**Fig. 6:**
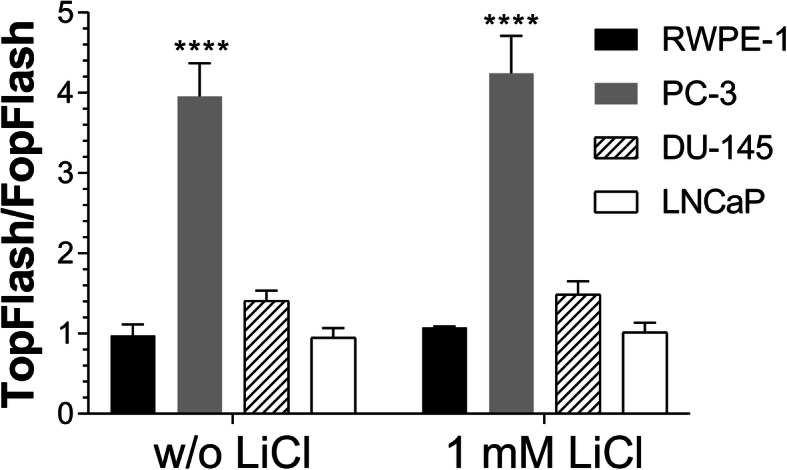
TOPFLASH TCF reporter assay. RWPE-1, PC-3, DU-145, and LNCaP cells were transfected with TOP or FOP plasmids and treated with or without 1 mM LiCl for 6 h. Luminescence levels are normalized to non-transfected cells. Data are plotted as the mean ± s.e.m., and four independent experiments were performed. Prostate cancer cells were compared to RWPE-1 cells using Dunnett’s test. **** *p* < 0.0001

To further verify whether the canonical Wnt pathway was being activated, the T-cell factor (TCF) reporter assay TOPFLASH was performed (Fig. 6). Interestingly, only PC-3 cells showed the increased activation of the canonical Wnt pathway compared with RWPE-1 cells.

### Wnt-3a binds to Glypican-1 and -5 in prostate cancer cells

**Fig. 7:**
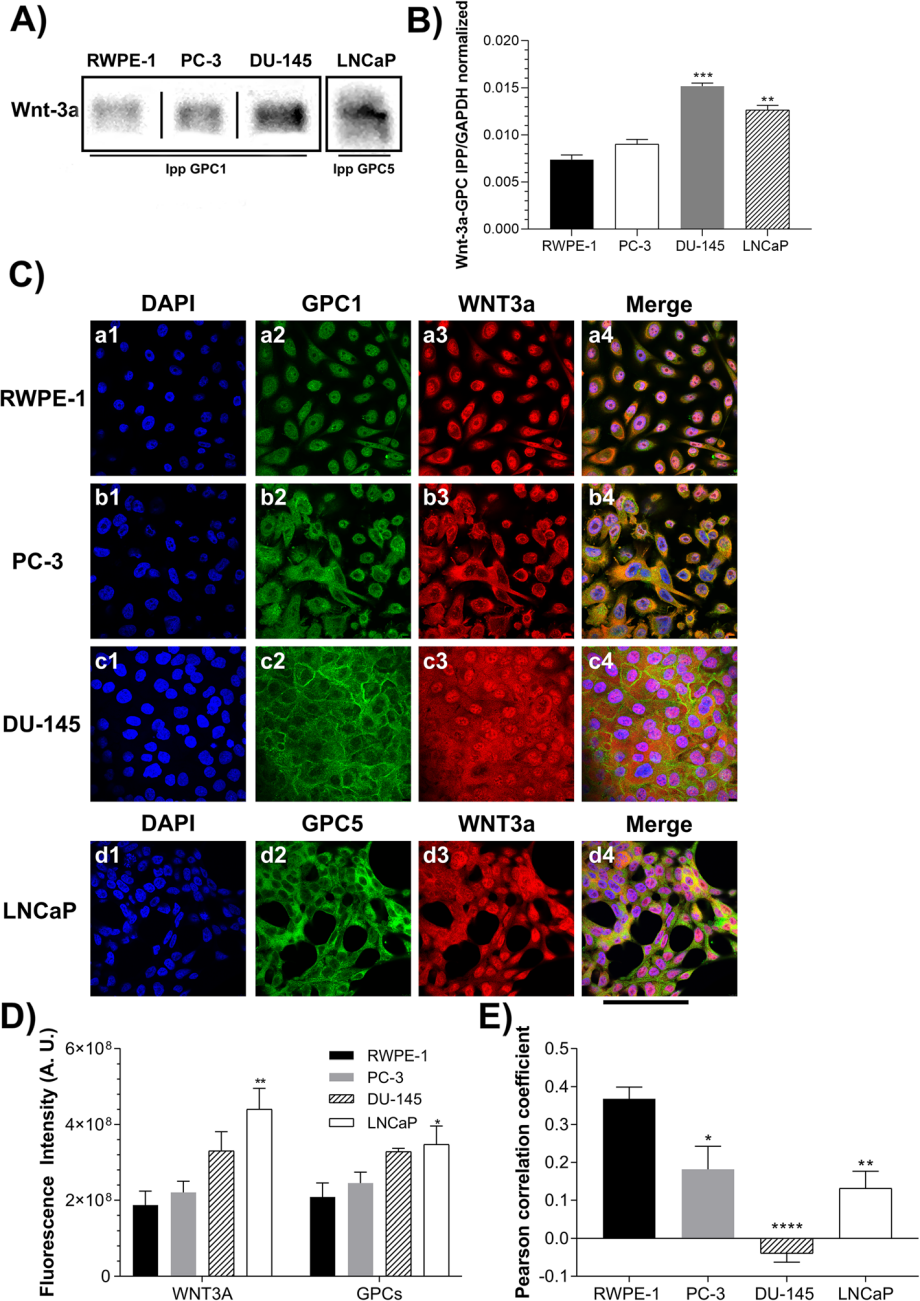
Analysis of the complex formation between GPCs and Wnt-3a

A co-immunoprecipitation assay was performed to establish whether a direct interaction occurs between Wnt-3a and the GPCs. The results demonstrated that an interaction between Wnt-3a and GPC1 was observed in RWPE-1, PC-3, and DU-145 cells; otherwise in LNCaP cells that interaction was observed between Wnt-3a and GPC5 (Fig. 7a). These interactions, which may modulate the canonical pathway, has been previously described by Capurro et al. between GPC3 and Wnt-3a [16]. Quantification by densitometric analysis showed higher levels of Wnt-3a linked to GPCs in prostate cancer cells than in normal cells, especially for DU-145 and LNCaP cells (Fig. 7b). We also performed an immunofluorescence assay to determine whether these molecules co-localize, and the results revealed that Wnt-3a and GPC1 co-localized to varying degrees in the examined cell types (Fig. 7c). Interestingly, using this technique, Wnt-3a and GPC1/GPC5 appeared to co-localize more frequently in RWPE-1 and PC-3 cells than in DU-145 and LNCaP cells (Fig. 7e). Furthermore, the co-localization observed in PC-3 and RWPE-1 cells appeared to concentrate more strongly at the cell surface. In contrast, in DU-145 and LNCaP cells, co-localization suggested to occur more intensely in the cytoplasm.

## Discussion

Suhovskih and colleagues detected GPC1 for the first time in prostate cancer in 2013, event that indicates the need of investigation regarding this molecule on the disease progression [17]. In our study GPC1 was the primary glypican gene expressed in PC-3, DU-145 and RWPE-1 cells, while GPC5 predominates in LNCaP cells. After assessing the different GPCs gene expression by RT-qPCR, we investigated the mainly expressed GPCs localization by confocal imaging. In PC-3, DU-145, and RWPE-1 cells, GPC1 was found at the outer region of the cell, suggesting a cell surface localization. In LNCaP cells, GPC5 appeared diffuse, possibly cytoplasmic. Early studies by Karthikeyan and colleagues (1994) demonstrated that HSPGs were mostly present at the plasma membrane, where they regulate the binding of growth factors, extracellular matrix proteins, and cell-cell adhesion molecules [18, 19]. Later, Mu and colleagues (2014) reported that GPC3 could be found both at the plasma membrane and in the cytoplasm of hepatocellular carcinoma cells [20]. However, the role of GPCs in the cytoplasm is not yet fully elucidated. In our study, we also detected reduced gene expression levels of GPC5 in PC-3 and DU-145 cells, as opposed to RWPE-1 cell line, which agrees with previously reported findings from breast and lung cancer studies [21–23]. These reports indicate that the lack of GPC5 would result in tumor progression, suggesting that this PG may play a role as a tumor suppressor.

Because the Wnt pathway is essential for cell adhesion, growth, and differentiation, its activation is crucial for tumor progression. β-catenin has a pivotal role in canonical Wnt signaling, and, as a transcription factor, after nuclear translocation, can mediate cellular events related to growth, apoptosis, migration, and adhesion [24]. Initially, to investigate the Wnt signaling pathway in prostate cancer cells, we analyzed the gene expression of β-catenin. The results indicated that in tumor cells (i.e., PC-3, DU-145 and LNCaP cells), the gene expression level of this protein is enhanced compared with normal prostate cells (i.e., RWPE cells, Fig. 3).

Our co-immunoprecipitation assays revealed the interactions between Wnt-3a and GPC-1 in PC-3, DU-145 and RWPE-1 cells; and Wnt-3a and GPC-5 in LNCaP cells. Previous studies demonstrated that GPCs are required for Wnt ligand binding. Our group has also previously shown the relevance of Wnt-3a to the prostate cancer progression, using PC-3 and DU-145 cell lines as a model [15]. In addition, reports from the literature already established the association of Wnt-3a with GPCs in several different types of cancers [25–28].

We showed through flow cytometry that prostate cancer cells had larger populations of Wnt-3a^+^ cells than RWPE-1 cells, and DU-145 cells presented a prevalent β-catenin^+^ population (Fig. 4). According to our immunoblotting assays (Fig. 5), we were also able to verify that both Wnt-3a and β-catenin were enhanced in prostate cancer cells when compared with normal cells. Interestingly, in the tumor cell lines (i.e., PC-3, DU-145 and LNCaP cells), β-catenin was more intensely identified to the nuclear compartment, when compared to RWPE-1 cells.

The translocation of β-catenin to the nucleus is associated with the activation of several genes intimately linked to prostate cancer. In the canonical pathway, Wnt-3a interacts with Frizzled and the LRP6/5 complex, resulting in the stabilization of cytosolic β-catenin and its translocation to the nucleus [29–31]. In the nucleus, β-catenin can associate with transcription factors, such as TCF/LEF-1, and promote the transcription of genes associated to cell proliferation and androgen receptor synthesis [32].

Although our results showed that higher amounts of β-catenin were present to the nuclear compartment in the tumor cell lines, especially in DU-145 and LNCaP cells, only PC-3 cells had activation of canonical Wnt signaling, as observed in the TOPFLASH TCF/LEF reporter assay. PC3 cells were also the only cells to display colocalization between Wnt-3a and GPC1 at the cell surface. It is of our belief that a GPC-WNT-Frizzled complex may occur in these instances [33].

The suggested cytoplasmic localization of Wnt-3a in DU-145 and LNCaP cells in this work, requires further studies. Interestingly, both DU-145 and LNCaP cells showed high levels of unphosphorylated β-catenin in both the nuclear and cytoplasmic compartments, and paradoxically TopFlash assay was not found activated in these cells. Nonetheless, this assay primarily detects transcription factor activation by TCFs. Although β-catenin may interact with TCF factors (e.g., TCF1, − 3, − 4 and LEF1), it may also cooperate with several others transcription factors, such as SOXs, FOXOs, HIF1α and MyoD [34]. Several SOX proteins, such as SOX2, − 3, 7 and − 17, repress β-catenin/TCF Wnt canonical activation [35]. SOX17 is able to halt epithelial to mesenchymal (EMT) transition (EMT) induced by canonical Wnt signaling [36]. The androgen nuclear receptor (AR) is a β-catenin interacting example, which in the presence of androgen increases this interaction and promotes nuclear accumulation of the complex [37–39]. Interestingly, studies have shown that androgen receptor may compete ligand-dependently with T-cell factor for nuclear β-catenin providing repression of the β-catenin/TCF signaling pathway [40, 41]. These data would explain to a certain extent, our findings in DU-145 and LNCaP cells, where absence of Wnt signaling activation was found, despite the interaction of Wnt-3a with GPCs. In any case, additional studies are required to clarify these issues.

The Wnt signaling pathway was also not activated in RWPE-1 cells, where GPCs and Wnt co-localized at the cell surface, event which requires further elucidation. Several studies have described GAG chains of the GPCs as being fundamental for interactions with other proteins, such as growth factors [42, 43]. Studies in the literature have revealed that GPC3 can bind to Wnt ligands by both the protein core [16, 44] as well as the heparan sulfate chains [45–47]. The role of the GAG chain modifications at the GPCs level could be determinant in the formation of GPC-Wnt-Frizzled complex [33]. In these cases, GPCs could function as a scavenge molecule for Wnt ligand, which may inhibit Frizzled receptor activation [48], and either the unavailability of Frizzled or impediment of the mentioned complexes, would disable WNT signaling. A clear comprehension of how GPCs interact with the Wnt cascade is fundamental, and efforts are being made to develop treatments that focus on HSPGs and Wnt signaling pathway components, which may assist prostate cancer patients in the future.

## Conclusions

GPC1 was the majorly expressed gene in all the studied cell lines, except for LNCaP, which expressed GPC5. We assessed by co-immunoprecipitation that these GPCs could interact with Wnt-3a. However, even though nuclear β-catenin was found increased in the prostate cancer cells (i.e., PC-3, DU-145 and LNCaP), activation of Wnt pathway was only found in PC-3 cells. In these PC-3 cells, GPC1 and Wnt-3a revealed high levels of colocalization, as assessed by confocal microscopy studies. This suggests a localization at the cellular membrane, where Frizzled receptor is required for downstream activation. The interaction of Wnt-3a with GPCs in DU-145 and LNCaP cells, which occurs in absence of Wnt signaling activation, requires further studies. Once non-TCF-LEF proteins can also bind β-catenin, another signaling pathway may be involved in these cells with regulatory function.

## Methods

### Materials

RPMI medium and penicillin/streptomycin were obtained from Sigma Chemical Co. (St. Louis, MO, USA). Keratinocyte-serum free medium was purchased from Thermo Scientific, Invitrogen (Waltham, MA, USA). Fetal bovine serum (FBS) was obtained from Cultilab (Campinas, Brazil). Polyclonal goat anti-glypican-1 (AF4519), polyclonal goat anti-glypican-5 (AF2607), monoclonal rat anti-Wnt-3a (MAB 9025), and monoclonal rabbit anti-β-catenin antibodies (MAB13291) were purchased from R&D Systems (Minneapolis, USA); monoclonal rabbit anti-GAPDH (ab128915) was purchased from ABCAM (Cambridge, MA, USA); polyclonal rabbit anti-lamin A (sc-20,680) was purchased from Santa Cruz Biotechnology (Dallas, TX, USA); and Alexa Fluor™594 Phalloidin (A12381) was obtained from Molecular Probes (Eugene, OR, USA). Anti-rat, anti-rabbit, anti-goat, and anti-mouse antibodies conjugated to Alexa Fluor 594, 488 or 633, and 4′,6-diamidino-2-phenylindole, dihydrochloride (DAPI) were all purchased from Molecular Probes.

### Cell culture

RWPE-1 (ATCC® CRL-11609™), PC-3 (ATCC® CRL-1435™), DU-145 (ATCC® HTB-81™) and LNCaP (ATCC® CRL-1740™) cells were purchased from the American Type Culture Collection (ATCC). RWPE-1 cells were cultivated in keratinocyte-serum free medium (K-SFM), supplemented with 0.05 mg/mL bovine pituitary extract and 5 ng/mL recombinant human epidermal growth factor (EGF). PC-3, DU-145, and LNCaP cell lines were cultivated in RPMI medium, supplemented with 10% FBS (v/v) and 100 μg/mL of penicillin and streptomycin. The cells were grown at 37 °C in a humidified atmosphere containing 5% CO_2_.

### Real-time quantitative PCR (RT-qPCR)

All procedures for qPCR adhered to the MIQE guidelines [49]. RWPE-1, PC-3, DU-145, and LNCaP cells were plated in 60-mm dishes, and total RNA was isolated according to manufacturer’s protocol using TRIzol™ reagent (Invitrogen). The reverse transcription reaction as well as the qPCR reaction were performed as previously described [50]. Table 1 shows the primers used in this assay.

**Table 1:** Primers used in RT-qPCR

Gene (human)	Forward and reverse primers (5′-NNN-3′)
GAPDH	F: 5′-TCGACAGTCAGCCGCATCTTCTTT-3′ R: 5′-ACCAAATCCGTTGACTCCGACCTT-3’
Glypican-1	F: 5’-TATTGCCGAAATGTGCTCAAGGGC-3′ R: 5′-ATGACACTCTCCACACCCGATGTA-3’
Glypican-2	F: 5’-TCCTTTCTGGTTCACACACTGGCT-3′ R: 5′-ACAGGCCATTGAATATGAGGGCGT-3’
Glypican-3	F: 5’-TGAAAGTGGAGACTGCGGTGATGA-3′ R: 5′-TCCCGAGGTTGTGAAAGGTGCTTA-3’
Glypican-4	F: 5’-TCGGAGATGTCCCTCGCAAATTGA-3′ R: 5′-TCTTCAACAGGGCATGGGTACACT-3’
Glypican-5	F: 5’-ACTGGCATGCATATATCCGGTCGT-3′ R: 5′-TGAGGTGAGCCTGTAACACAGCAT-3’
Glypican-6	F: 5’-CTTCATTGCTGCCAGGACCTTTGT-3′ R: 5′-AGCCCTTCATGACGTTGAGACAGT-3’
β-catenin	F: 5’-CTTCACCTGACAGATCCAAGTC-3′ R: 5′-CCTTCCATCCCTTCCTGTTTAG-3’

The genes expressions were compared using a relative quantitation method with 2^-∆Ct^. ∆Ct for each replicate/gene was obtained by subtracting the Ct value from the Ct average of GAPDH (i.e., the housekeeping gene). The value of 2^-∆Ct^ was then obtained.

### Flow cytometry assay

RWPE-1, PC-3, DU-145, and LNCaP cells were plated at a density of 2 × 10^6^ cell/mL and incubated for 48 h. The cells were then detached, fixed and permeabilized as described [50], followed by PBS washing. Anti-glypican-1 (goat, 1:100), anti-glypican-5 (goat, 1:100) or anti-β-catenin (rabbit, 1:100) and anti-Wnt-3a (rat, 1:100) antibodies were diluted in 1% bovine serum albumin (BSA) in PBS, and 50 μL of the combined antibody solution was incubated with the cells for 2 h. Subsequent PBS washing step was followed by addition of 50 μL of the secondary antibody solution containing anti-rat Alexa Fluor 488 (1:200) and anti-goat Alexa Fluor 633 (1:200) and incubated for an additional 40 min. The cells were collected in 400 μL PBS and assessed using a BD FACSCalibur (Becton Dickinson, CA, USA).

### Immunofluorescence and confocal imaging

RWPE-1, PC-3, DU-145, and LNCaP cells were plated (2 × 10^4^ cells/well) onto coverslips. The well contents were PBS washed and fixed with cold 2% paraformaldehyde for 30 min. The cells were washed with 0.1 M glycine, and then blocked and permeabilized with 0.01% saponin and 5% FBS/PBS for 15 min. Anti-glypican-1 (goat, 1:100), anti-glypican-5 (goat, 1:100), or anti-β-catenin (rabbit, 1:100) antibodies were diluted in 5% FBS in PBS and incubated for 2 h at room temperature. The coverslips were washed three times with PBS and incubated for 1 h with anti-goat or anti-rabbit Alexa Fluor 594 (1:200) diluted in PBS. The cells were washed five times and incubated with a 1:1500 (v/v) DAPI solution containing 0.01% of saponin for 30 min. The coverslips were washed six times with PBS, dipped in cold type I water, mounted with a Fluoromount™ solution (2:1 in PBS) onto clean slides, and sealed with colorless nail polish. Confocal imaging was performed using Zeiss Confocal Microscope Observer Z.1 (Zeiss Carl, Jena, Germany).

For co-localization assays between glypican-1 or − 5 and Wnt-3a, cells were washed in cold PBS and fixed in pre-warmed 4% paraformaldehyde for 15 min. The cells were washed with 0.1 M glycine and then were blocked for 1 h in PBS containing 0.1% Triton X-100 and 3% BSA. Coverslips were then incubated with anti-glypican-1 (goat, 1:100) or anti-glypican-5 (goat, 1:100), and anti-Wnt-3a (mouse, 1:100) antibodies diluted in PBS containing 0.05% Triton X-100 and 1% BSA, overnight at 4 °C. After three washes in PBS, coverslips were incubated for 1 h with anti-goat Alexa Fluor 488 (1:200) and anti-mouse Alexa Fluor 647 (1:200) secondary antibodies in PBS containing 0.05% Triton X-100 and 1% BSA. The cells were washed three times with PBS, incubated with DAPI (1:2000) in PBS for 15 min, and washed five times with PBS before being mounted onto clean slides with Fluoromount™. Images were captured using a Leica confocal microscope, TCS SP8.

### Immunoblotting assay

RWPE-1, PC-3, DU-145, and LNCaP cells were grown in 100-mm Petri dishes until they reached 85% confluence. Then the cells were lysed with 1 mL lysis buffer (100 mM NaCl, 10% glycerol, and 10% Sigma Aldrich protease and phosphatase inhibitor cocktail in 20 mM Tris-HCl, pH 8.0) and agitated for 30 min at 4 °C. The protein extract was obtained from the supernatant, following centrifugation at 12,000 rpm for 20 min.

An alternative procedure was performed to obtain cytoplasmic and nuclear fractions. In brief, 85% confluent cells were washed with cold PBS and lysed with 500 μL lysis buffer (10 mM Tris, pH 7.4, 10 mM NaCl, 3 mM MgCl_2_, 0.5% NP-40, 50 mM NaF, 2 mM Na_3_VO_4_, 10 mM Na_4_P_2_O_7_, 1 mM PMSF, 10 μg/mL leupeptin, and 10 μg/mL aprotinin), for 20 min at 4 °C. Then, 700 μL lysis buffer containing an additional 1 M sucrose was added and centrifuged at 3600 rpm for 10 min. The supernatant, containing the cytoplasmic extract, was move to a new tube, and the precipitate was resuspended in 100 μL nuclear extraction buffer (200 mM NaCl, 10 mM HEPES, pH 7.9, 1.5 mM MgCl_2_, 0.1 mM EDTA, 5% glycerol, 50 mM NaF, 2 mM Na_3_VO_4_, 10 mM Na_4_P2O_7_, 1 mM PMSF, 10 μg/mL leupeptin, and 10 μg/mL aprotinin) and incubated for 15 min, at 4 °C, under continuous agitation. The samples were centrifuged at 10,400 rpm for 30 min, and the supernatant containing the nuclear extract was moved to a new tube. The protein concentration was determined using the BCA protein assay kit (Pierce, Thermo Fisher Scientific, USA).

Fifty micrograms of the cell protein extract, or 30 μg of either the cytoplasmic or nuclear extracts, were diluted in sample buffer (4% SDS, 0.02% bromophenol blue, 20% glycerol, and 200 mM DTT in 100 mM Tris-HCl, pH 6.8) and boiled for 5 min. The samples were applied to a 10% polyacrylamide gel, which was subjected to electrophoresis (100 V for 2 h). The separated proteins were transferred onto nitrocellulose membranes (400 mA for 1 h) in a 4% methanol, 200 mM glycine, and 25 mM Tris-containing buffer. The membranes were blocked with 2.5% skimmed milk in TBS with 0.1% of TWEEN20® (TBS-T) for 2 h at 4 °C, and then incubated with anti-Wnt-3a (rat, 1:1000), anti-β-catenin (rabbit, 1:1000), anti-lamin A (rabbit, 1:1000) or anti-GAPDH (rabbit, 1:1000) antibodies diluted in 1% BSA in TBS-T overnight at 4 °C. The membranes were washed with TBS-T and incubated with anti-rat or anti-rabbit horseradish peroxidase (HRP) conjugated secondary antibodies (1:2000) diluted in 1% BSA in TBS-T for 2 h. The membranes were then washed with TBS and revealed with a chemiluminescent substrate (Supersignal, Sigma) using the MF-ChemiBIS Bio-Imaging System. Densitometric analysis was performed using ImageJ software (National Institutes of Health, USA).

### Protein immunoprecipitation

After protein extraction, described in section 5.6, 500 μg total protein was incubated with anti-glypican-1 (goat 1:50) or anti-glypican-5 (goat, 1:50) antibody in lysis buffer, overnight at 4 °C, under continuous agitation. Then, 50 μL of protein A/G plus-agarose beads (Santa Cruz) were added to the samples and incubated at 4 °C for 4 h, under continuous agitation. The beads were precipitated by centrifugation (5000 rpm for 5 min), washed three times with 1 mL PBS, and washed twice with 1 mL 20 mM Tris-HCl, pH 8.0. The final pellet was resuspended in sample buffer and boiled for 5 min. Wnt-3a was assessed from GPC pull-downs by immunoblot.

### TOPFLASH TCF reporter assay

RWPE-1, PC-3, DU-145, and LNCAP cells were plated at a density of 3 × 10^4^ cells/well in a 96-well white plate. The following day, the medium was changed to OptiMEM (Gibco®, Thermo Fisher Scientific). Then the cells were transfected with the TCF firefly luciferase reporters (pTOPFLASH, TCF Optimal motif, and pFOPFLASH, Far from Optimal motif) [51], using Fugene HD (Promega, Wisconsin, US) in a 4:1 proportion by applying 10 μL transfection medium to each well. Untransfected cells were used as a control group. After 18 h, the medium was changed, and 1 mM LiCl was added to half of the cells and incubated for 6 h. Luminescence was measured using SteadyLitePlus™ (Perkin Elmer, Massachusetts, MA. US) according to the manufacturer’s instructions. The results were calculated as a TOPFLASH/FOPFLASH ratio, to exclude bias due to cell death or proliferation following treatments.

### Statistical analysis

Data were analyzed using GraphPad Prism 8.0 and Microsoft Office Excel 2016. Data were analyzed using one-way analyses of variance (ANOVA) with Dunnett’s test. The comparisons performed were between the effects observed in the prostate cancer cell lines (PC-3, DU-145, and LNCaP) and the normal prostate cell line (RWPE-1). Statistically significant comparisons were assessed at the 5% level, and data were plotted as the mean ± standard error of the mean (s.e.m.).

## Supplementary Information

**Additional file 1: Figure S1.** Uncropped immunoblot membranes concerning Fig. 5a.

**Additional file 2: Figure S2.** Uncropped immunoblot membranes concerning Fig. 7a

## Data Availability

The datasets generated from this study are available from the corresponding author on reasonable request.

## Abbreviations

ANOVA: Analysis of variance
APC: Adenomatous polyposis coli protein
AR: Androgen receptor
ATCC: American type culture collection
BMP: Bone morphogenetic protein
BSA: Bovine serum albumin
CK1: Casein kinase 1
DAPI: 4′,6-diamidino-2-phenylindole, dihydrochloride
EGF: Epidermal growth factor
EMT: Epithelial to mesenchymal transition
FBS: Fetal bovine serum
FGF: Fibroblast growth factor
GAG: Glycosaminoglycan
GAPDH: Glyceraldehyde-3-phosphate dehydrogenase
GPC: Glypican
GPI: Glycosylphosphatidylinositol
GSK3: Glycogen kinase 3
Hh: Hedgehog
HRP: Horseradish peroxidase
HS: Heparan sulfate
HSPG: Heparan sulfate proteoglycan
K-SFM: Keratinocyte-serum free medium
LDL: Low-density liporeceptor
LEF: Lymphoid enhancer binding factor
LRP5/6: Receptor related protein 5/6
PG: Proteoglycan
PSA: Prostate-specific antigen
RT-qPCR: Reverse transcription real-time quantitative PCR
s.e.m.: Standard error of the mean
TCF: T-cell factor
TGF: Transforming growth factor
