# Supplementary figures and images for: The Glypican proteoglycans show intrinsic interactions with Wnt-3a in human prostate cancer cells that are not always associated with cascade activation
Source: BMC Mol Cell Biol. 2021 May 4;22:26. doi: 10.1186/s12860-021-00361-x (PMC8097805; doi:10.1186/s12860-021-00361-x)

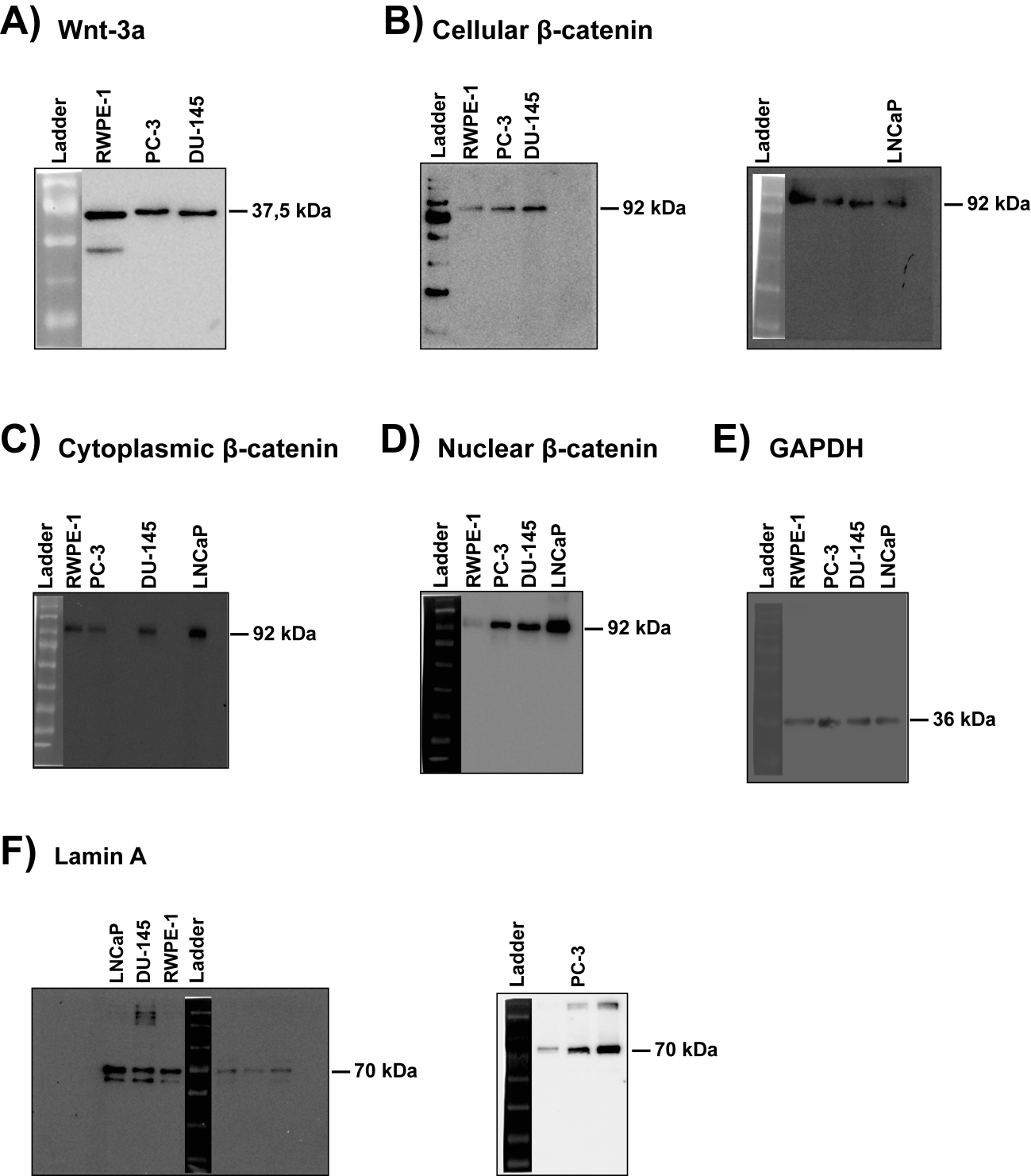


**Figure S1. Uncropped immunoblot membranes concerning Figure 5A**.

Supplement: Supplementary file 1 — Additional file 1: Figure S1. Uncropped immunoblot membranes concerning Fig. 5a. [file 12860_2021_361_MOESM1_ESM.docx]

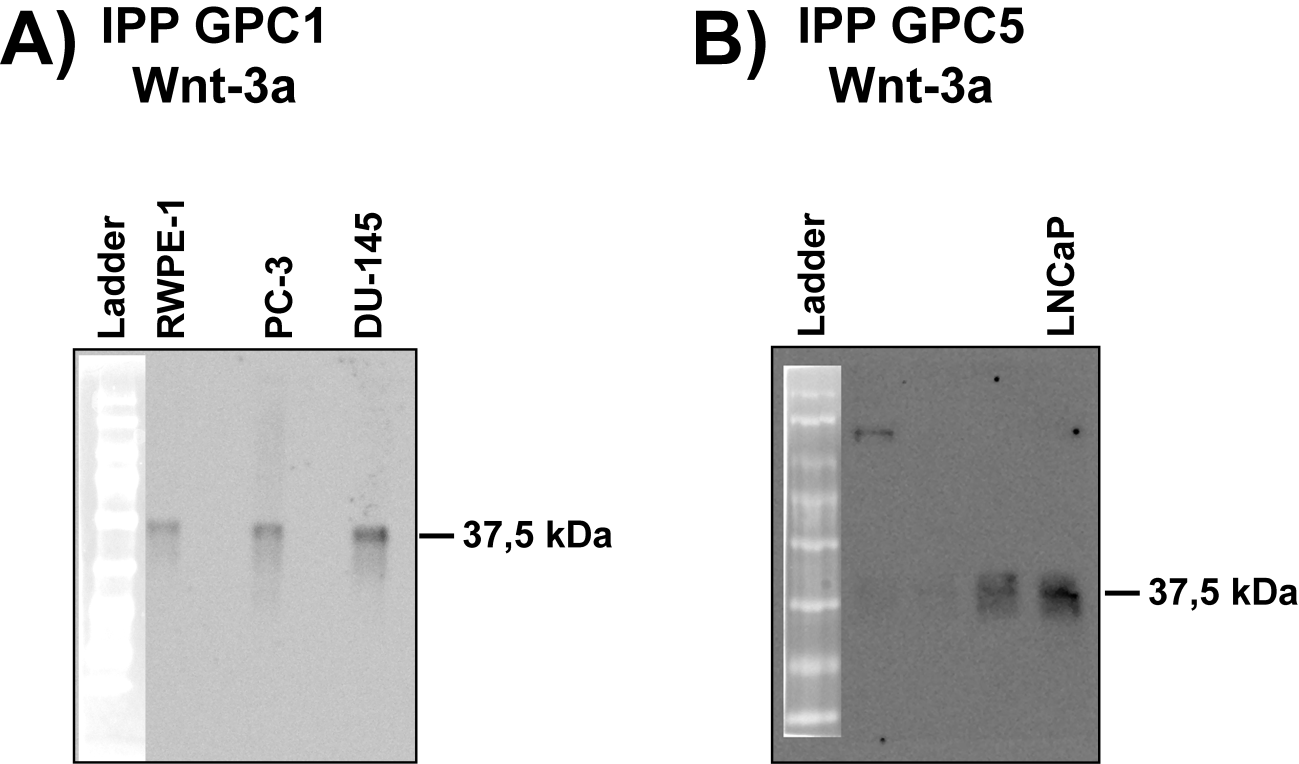


**Figure S2. Uncropped immunoblot membranes concerning Figure 7A**.

Supplement: Supplementary file 2 — Additional file 2: Figure S2. Uncropped immunoblot membranes concerning Fig. 7a [file 12860_2021_361_MOESM2_ESM.docx]
